# Enantioselective Cycloaddition Reactions Catalyzed by BINOL-Derived Phosphoric Acids and *N*-Triflyl Phosphoramides: Recent Advances

**DOI:** 10.3390/molecules200916103

**Published:** 2015-09-03

**Authors:** Felix E. Held, Dominik Grau, Svetlana B. Tsogoeva

**Affiliations:** Department of Chemistry and Pharmacy, Organic Chemistry Chair I and Interdisciplinary Center for Molecular Materials, University of Erlangen-Nürnberg, Henkestrasse 42, Erlangen 91054, Germany; E-Mails: felix.held@fau.de (F.E.H.); dominik.grau@fau.de (D.G.)

**Keywords:** cycloaddition reactions, BINOL-derived phosphoric acids, *N*-triflyl phosphoramides, enantioselective pericyclic reactions

## Abstract

Over the last several years there has been a huge increase in the development and applications of new efficient organocatalysts for enantioselective pericyclic reactions, which represent one of the most powerful types of organic transformations. Among these processes are cycloaddition reactions (e.g., [3+2]; formal [3+3]; [4+2]; vinylogous [4+2] and 1,3-dipolar cycloadditions), which belong to the most utilized reactions in organic synthesis of complex nitrogen- and oxygen-containing heterocyclic molecules. This review presents the breakthrough realized in this field using chiral BINOL-derived phosphoric acids and *N*-triflyl phosphoramide organocatalysts.

## 1. Introduction

**Figure 1:**
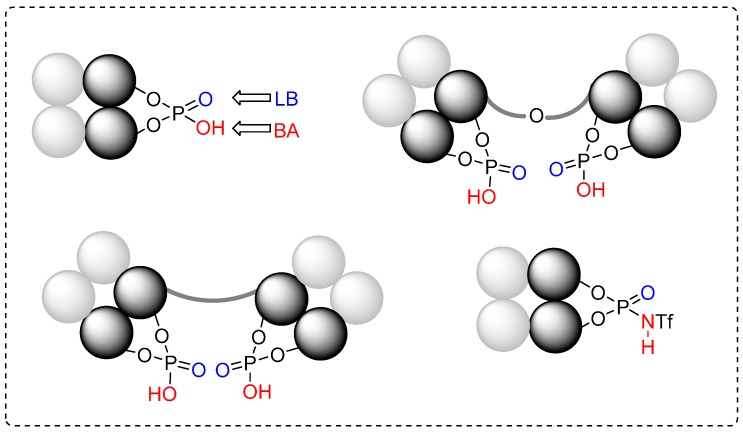
Schematic presentation of bifunctional organocatalysts discussed in this article. LB = Lewis Base; BA = Brønsted Acid.

The enantioselective Diels-Alder reaction is one of the most important and powerful pericyclic reactions for the synthesis of complex molecules [1,2,3]. This [4+2] and related cycloaddition reactions (e.g., [3+2]; formal [3+3]; vinylogous [4+2] and 1,3-dipolar cycloadditions) provide straightforward access to versatile chiral carbo- and heterocyclic compounds. As a result, extensive research effort has been dedicated to the development of chiral organocatalysts for highly stereo- and regioselective versions of different cycloaddition transformations. Many excellent results have been achieved by applying BINOL-derived phosphoric acids and *N*-triflyl phosphoramides. In this review, we discuss recent achievements in developing enantioselective cycloaddition transformations using bifunctional chiral phosphoric acids and *N*-triflyl phosphoramides with the general structures given in Figure 1.

After a corresponding short overview of the development of the selected organocatalysts, different cycloaddition reactions and their formal versions ([3+2]; formal [3+3]; [4+2]; vinylogous [4+2] and 1,3-dipolar cycloadditions), catalyzed by these powerful organocatalysts, will be described in this review article.

## 2. Cycloadditions Catalyzed by Chiral BINOL-Derived Phosphoric Acids

Since the pioneering studies of the groups of Akiyama [4] and Terada [5] in 2004 on the application of chiral BINOL-derived phosphoric acids as powerful Brønsted acid catalysts in different C-C bond formation reactions, the development of novel BINOL-phosphate catalyzed reactions has been continuously studied and resulted in great progress in recent years [6,7,8,9,10,11].

Several research groups have already reported the application of BINOL-phosphates in numerous highly enantioselective transformations [6,7,8,9,10,11]. In most cases the key aspect of catalysis is the bifunctional character (Brønsted acid/Lewis base) of the phosphoric acid moiety. Asymmetric catalysis in the cycloaddition reactions (e.g., [3+2], formal [3+3], [4+2], vinylogous [4+2] and 1,3-dipolar cycloadditions) has also been successfully realized using chiral Brønsted-acid catalysts and opened up a promising new frontier in organic synthesis.

As such, BINOL-phosphate catalyzed [3+2] cycloadditions have been introduced as a convenient direct method for the synthesis of pyrazolidine heterocycles [12,13,14], which are ubiquitous in pharmaceutical compounds with antitumor, antimicrobial, anticonvulsant and other biological activities.

Furthermore, recent asymmetric [4+2], formal [3+3] and 1,3-dipolar cycloadditions developed by other research groups represent facile methods for the enantioselective construction of several versatile heterocyclic core structures (e.g., six-membered piperidine frameworks [15], 4-aminobenzopyrans [16], tetrahydrocarbazoles [17], 3-methylenepyrrolidines [18], hexahydrochromeno[4,3-*b*]pyrrolidines [19], spirocyclic oxindole derivatives [20,21] and spiro[pyrazolidin-3,3′-oxindoles] [22]) widely present in numerous bioactive compounds and natural products.

### 2.1. [3+2] Cycloadditions

In their continuous efforts to develop new efficient procedures for the synthesis of bioactive nitrogen-containing heterocycles [23], the Tsogoeva group became interested in the stereoselective synthesis of pyrazolidines by reliable cycloaddition reactions. As a continuation of their research in developing organocatalytic approaches to reactions involving *N*-acylhydrazones [24,25,26] as readily available and stable reactants, they initiated the investigation of the cycloaddition reaction, which could constitute a first catalytic metal-free intermolecular [3+2] cycloaddition of different *N*-acylhydrazones to dienes providing pyrazolidines.

**Scheme 1:**
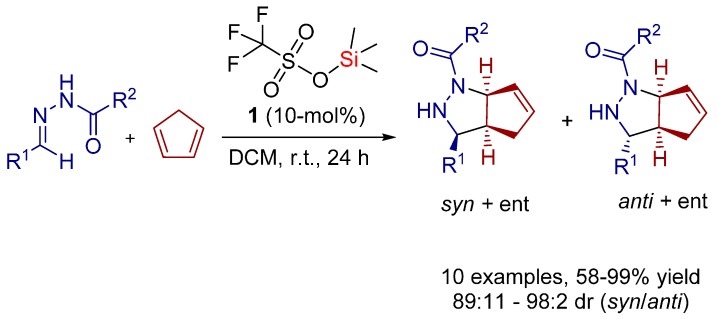
[3+2] Cycloaddition reaction between *N*-acylhydrazones and cyclopentadiene catalyzed by TMSOTf (trimethylsilyl triflate, **1**).

As a result, in 2011, Tsogoeva and co-workers demonstrated for the first time that the [3+2] cycloaddition reaction between *N*-acylhydrazones and cyclopentadiene can be successfully performed in high yields (up to 99%), with diastereoselectivity up to 98:2 dr using catalytic amounts of TMSOTf (trimethylsilyl triflate, **1**) as readily available achiral silicon Lewis acid catalyst (Scheme 1) [12,14].

**Scheme 2:**
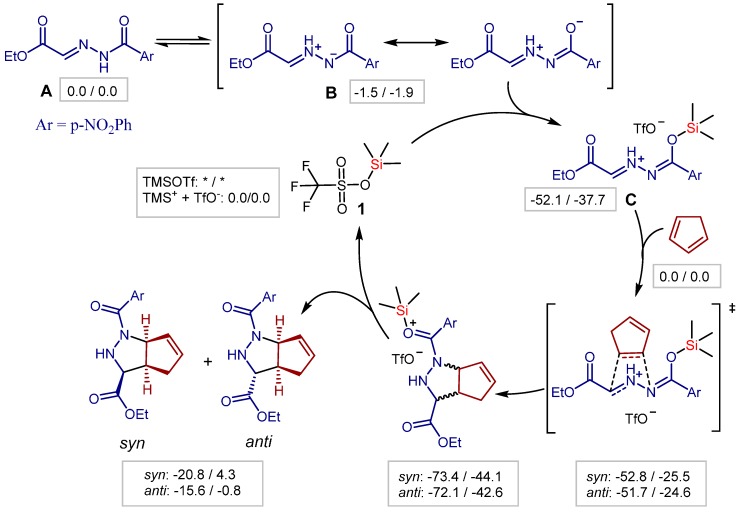
Mechanistic studies at density functional theory (DFT) level—B3LYP/TZV (energies in frames: ΔH/ΔG in kcal/mol); * the dissociation energy of TMSOTf (trimethylsilyl triflate, **1**) is overestimated; TMS^+^ + TfO^−^ is used as reference state.

Additionally to the experimental findings, DFT (density functional theory) calculations on the reaction mechanism were carried out (Scheme 2).

It was found that the hydrazone isomer **B** is stabilized relative to **A**. The DFT results suggest that, once **B** emerges, a complex **C** with the Lewis acidic silicon center is formed. Since the triflate is an excellent leaving group, it is eliminated when the catalyst approaches. From this point, the calculations suggest a plausible mechanistic pathway, which is based on the formation of a stable Si-O-bond.

Nonetheless, identification of an enantioselective metal-free catalyst system for this useful intermolecular reaction remained an important challenge at that time.

**Figure 2:**
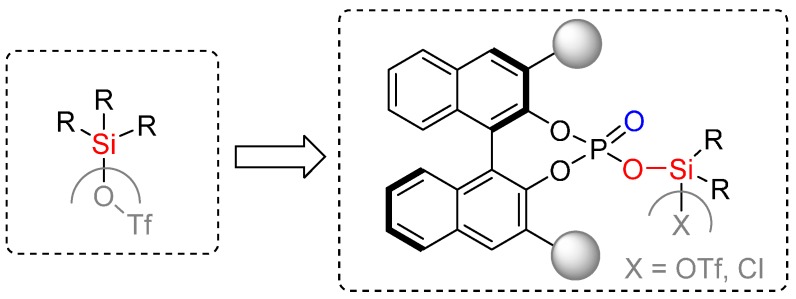
Design of new chiral catalyst for [3+2] cycloaddition reaction.

Towards this goal, Tsogoeva and co-workers envisioned the achievement of an enantioselective intermolecular [3+2] cycloaddition of *N*-acylhydrazones to olefins by combining an achiral silicon Lewis acid catalyst with chiral BINOL-phosphates (Figure 2).

**Scheme 3:**
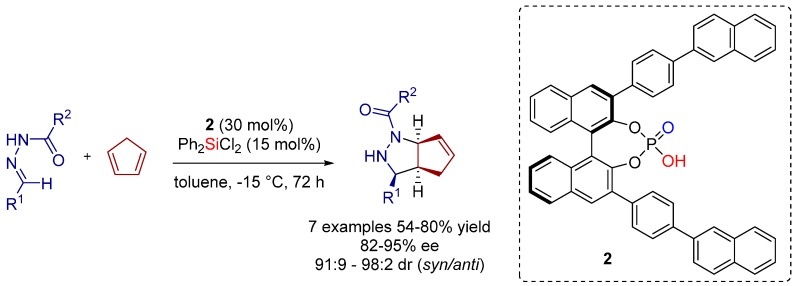
Enantioselective [3+2] cycloaddition reaction catalyzed by a combination of chiral BINOL-phosphate with achiral silicon compound.

**Figure 3:**
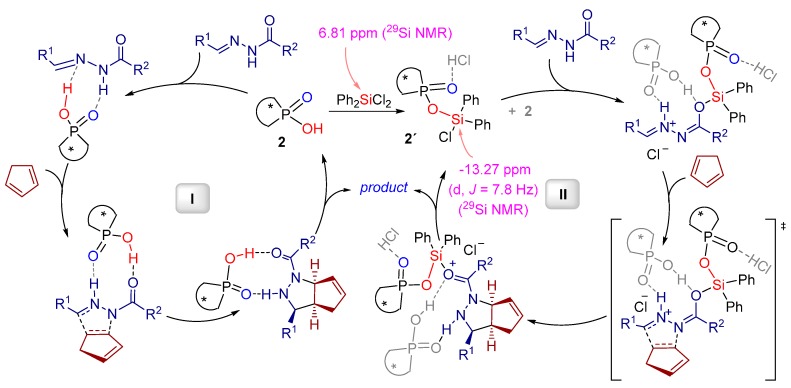
Proposed mechanisms for [3+2] cycloaddition reaction by using BINOL-phosphate alone (I) and applying BINOL-phosphate/Ph_2_SiCl_2_ (2:1) as a catalytic system (II).

Therefore, in 2012 the Tsogoeva group further reported an enantioselective [3+2] cycloaddition as a convenient method towards chiral pyrazolidines in good yields and with high levels of dia- and enantioselectivities, utilizing a cooperative catalytic system of BINOL-derived phosphoric acid **2** and an *in situ* generated BINOL-phosphate-derived silicon Lewis acid (Scheme 3, Figure 3) [13].

Notably, the weak Lewis acid additive Ph_2_SiCl_2_ alone was inactive in the catalysis under these reaction conditions. Also BINOL-phosphate itself showed low reactivity and moderate enantioselectivity (13% yield, 47% ee, 99:1 dr). Interestingly, the combination of both components resulted in very good reaction outcome (Scheme 3), implying the *in situ* activation of a weak Si-Lewis acid (Ph_2_SiCl_2_) by connection to a strongly electron-withdrawing group, which is represented by the BINOL-derived phosphoric acid moiety. The assumption that the presence of the leaving group in the *in situ* generated catalyst (Figure 3) is required for efficient catalysis is supported by the fact that no reaction occurs using the combination of **2** with Ph_3_SiCl.

Based on the previous DFT calculations [12] and ^29^Si-NMR studies two independent catalytic cycles were proposed by the authors. As it has already been noted, BINOL-phosphate alone is sufficient to generate the desired products (Figure 3, cycle **I**), although, with only moderate results. Cooperative silicon-Lewis acid/Brønsted acid catalysis, therefore, has been suggested to be more efficient (Figure 3, cycle **II**). The Lewis basic phosphoryl oxygen in cycle **II** might capture the HCl, generated through the *in situ* formation of *O*-silylated BINOL-phosphate species and, hence, inhibit competitive non-enantioselective catalysis by the achiral Brønsted acid. The additional molecule of chiral Brønsted acid **2** likely coordinates to the hydrazone via hydrogen bonds stabilizing the proposed transition state structure.

### 2.2. Formal [3+3] Cycloadditions

Chiral six-membered heterocycles are present as core structures in natural alkaloids and bioactive molecules. Due to their significant importance, the development of a convenient preparation method is a challenging task for organic chemists. An elegant way towards these compounds is the catalytic asymmetric azomethine ylide-involved cycloaddition which, however, has only been investigated very sparsely to date.

**Scheme 4:**
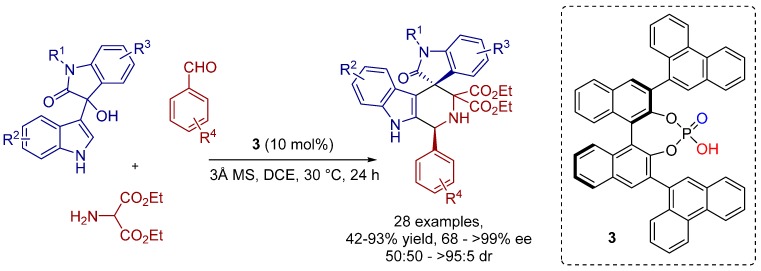
Asymmetric formal [3+3] cycloaddition of isatin-derived 3-indolylmethanols and an *in situ* generated azomethine ylide.

In this context, the high potential of BINOL-phosphate catalysts for the asymmetric formal [3+3] cycloaddition of an *in situ* generated azomethine ylide with isatin-derived 3-indolylmethanol towards chiral six-membered piperidine frameworks has been recently demonstrated by Shi, Tu and co-workers (Scheme 4) [15].

The generality of this cycloaddition method was proven by construction of structurally diverse spiro[indoline-3,4′-pyridoindoles] with good to high yields and excellent enantioselectivities in most cases. Nonetheless, it was evident that the electronic nature of the aldehydes had an influence on the stereoselectivity of the reaction.

**Figure 4:**
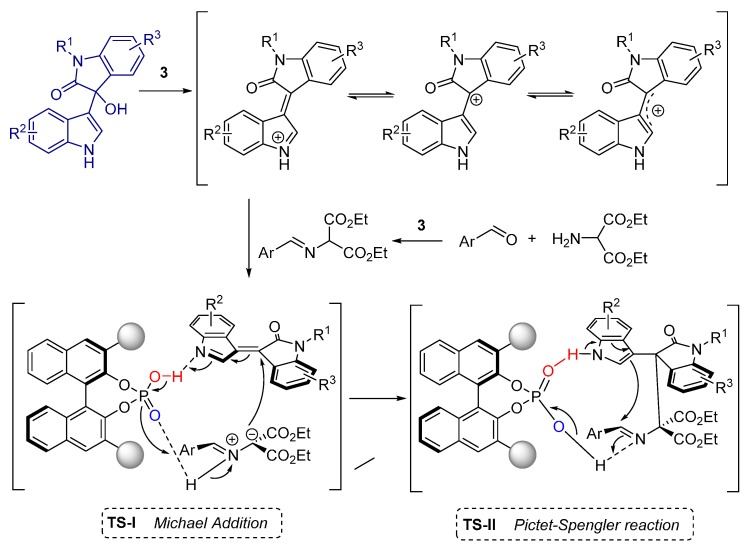
Proposed reaction mechanism and transition state structures.

The authors proposed the reaction mechanism and related transition state structures to describe the stereochemistry of the formal [3+3] cycloaddition (Figure 4). The reaction might proceed via a sequential Michael addition (TS-I) and Pictet-Spengler reaction (TS-II), whereby the chiral phosphoric acid simultaneously activates both vinyliminium intermediate and azomethine ylide and, furthermore, the chiral environment is created through the bulky substituents of the catalyst.

### 2.3. [4+2] and Vinylogous [4+2] Cycloadditions

**Scheme 5:**
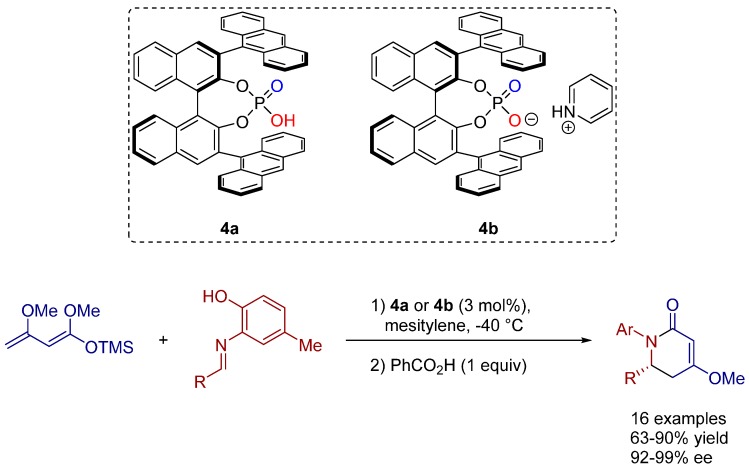
Asymmetric Diels-Alder reaction of Brassard’s diene and imines.

Being of great interest for the pharmaceutical industry, piperidine derivatives are important synthetic targets as precursors for the biologically significant piperidine alkaloids, peptides, and aza sugars. In 2006 Akiyama and co-workers successfully developed a first highly enantioselective aza-Diels-Alder reaction of Brassard’s diene and imines, promoted by a chiral Brønsted acid catalyst to furnish dihydropyridone derivatives in high yields and enantioselectivities (Scheme 5) [27].

The catalytic system has shown high enantioselectivity for the reaction of a wide range of imine substrates with good functional group tolerance. Furthermore, the authors observed a significant increase of the yield by substitution of the chiral Brønsted acid catalyst **4a** with its corresponding pyridinium salt **4b**. Further NMR studies concerning the stability of Brassard’s diene in the presence of catalyst **4a** revealed that this phenomenon correlates with the decomposition of the rather labile diene by the stronger acidic Brønsted acid catalyst compared to its pyridinium salt.

**Figure 5:**
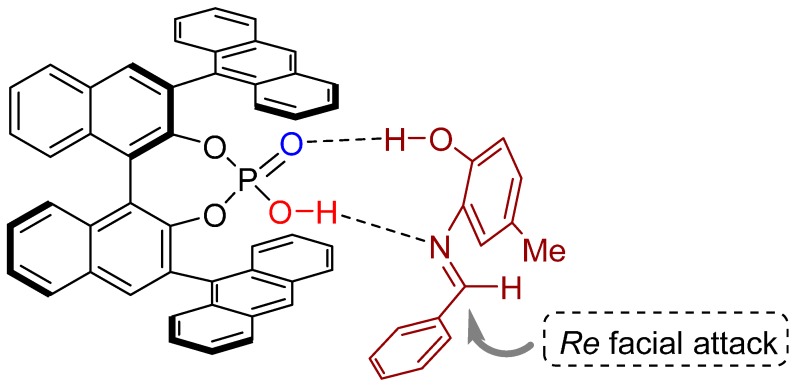
Proposed nine-membered cyclic transition state; the arrow indicates *Re* facial attack.

A mechanistic explanation was proposed to account for the high enantioselectivity of the Diels-Alder reaction described above. Based on mechanistic studies, the authors propose that the reaction proceeds via a nine-membered cyclic transition state which involves the hydrogen atom of the hydroxy group, demonstrating the significance of this moiety on the *N*-aryl group. Thus, the attack of the nucleophile is directed towards the *Re*-face of the aldimine (Figure 5).

**Scheme 6:**
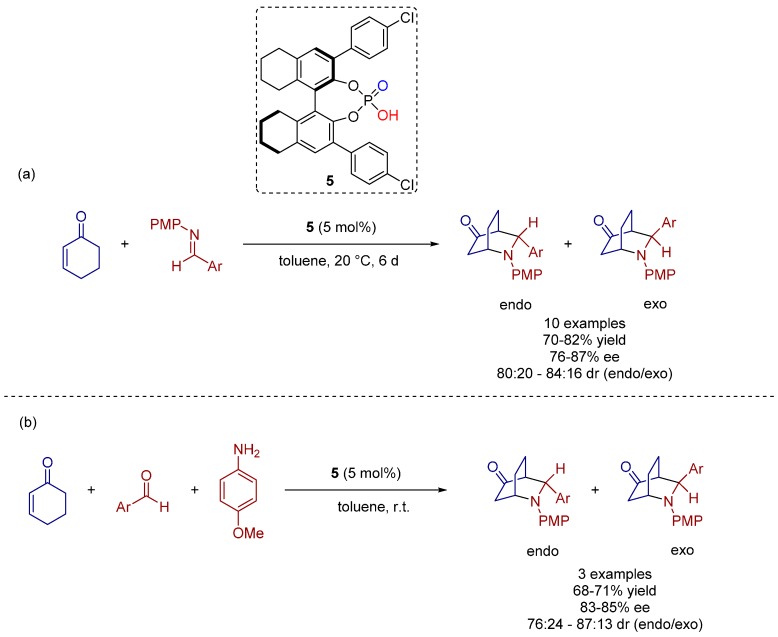
(**a**) Organocatalytic asymmetric Diels-Alder reaction of cyclohexanone with aromatic aldimines; (**b**) one-pot three-component asymmetric aza-Diels-Alder reaction.

In the same year, Gong and co-workers presented an alternative route towards chiral substituted piperidines within the first chiral Brønsted acid catalyzed asymmetric direct aza-Diels-Alder reaction of aromatic aldimines with cyclohexanone furnishing the products with good yields and enantioselectivities (Scheme 6) [28].

The scope of this methodology was successfully extended to other benzaldimines, affording preferably the endo-isomer of the corresponding cycloadducts in good yields and enantioselectivities and, hence, convenient access to a wide range of *N*-containing heterocyclic compounds is provided (Scheme 6a).

Moreover, the reaction could be successfully carried out in a one-pot three-component manner without any loss of enantioselectivity (Scheme 6b).

**Figure 6:**
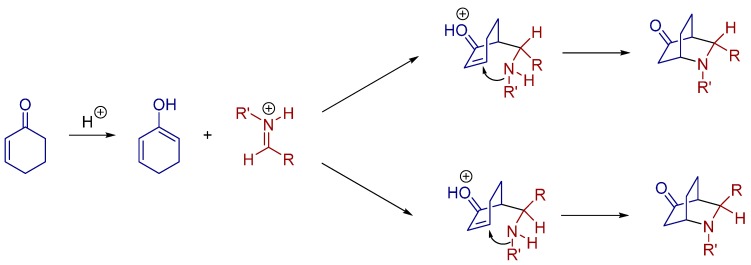
Proposed direct aza-Diels-Alder reaction of cyclohexanone with aldimines.

Furthermore, these authors proposed a possible mechanism, as outlined in Figure 6. At first, cyclohexenone is enolized, undergoing a Mannich reaction with the protonated aldimine, which has been activated by the chiral phosphoric acid catalyst. Subsequently, the final cycloaddition products are generated through a tandem intramolecular 1,4-addition reaction (Figure 6).

**Scheme 7:**
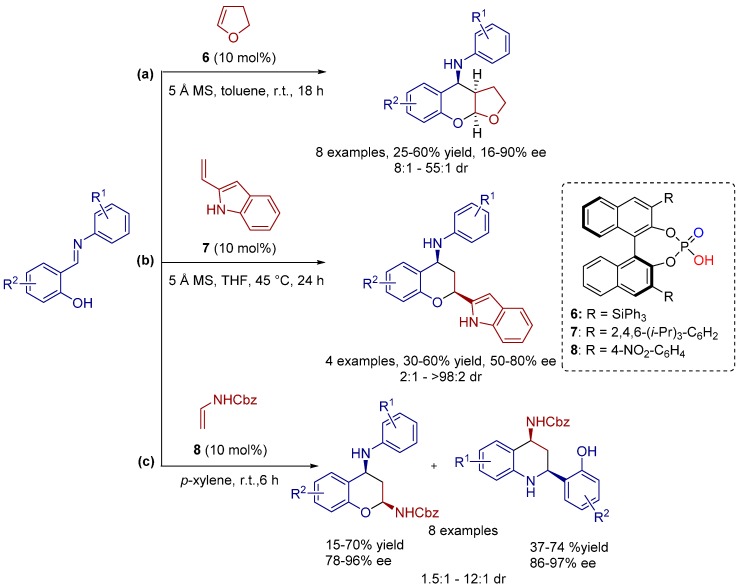
Inverse-electron-demand [4+2] cycloadditions of *N*-phenylsalicylaldimines with (**a**) 2,3-dihydro-*2H*-furan; (**b**) 2-vinylindole and (**c**) *N*-vinylcarbomate.

The 4-aminobenzopyrans and their furan-fused derivatives appear to be very appealing targets for synthetic chemists due to their useful biological properties such as anti-hypertensive and anti-ischaemic activities. In 2010, Fochi and co-workers developed the BINOL-phosphate catalyzed inverse-electron-demand [4+2] cycloaddition reactions of different salicylaldehyde-derived *N*-arylimines with electron-rich dienophiles such as 2,3-dihydro-2*H*-furan, 2-vinylindole and *N*-vinylcarbamate towards chiral 4-aminobenzopyran derivatives (Scheme 7) [16].

Chemoselectivity strongly relied on the nature of the compound employed as the electron-rich dienophile. As substrates 2,3-dihydro-2*H*-furan and 2-vinylindole, for instance, generate only 4-aminobenzopyrans, whereas *N*-vinylcarbamate undergoes also a [4+2] aza-Diels-Alder cycloaddition (Povarov reaction) with the *N*-arylimine moiety of salicylaldehyde-derived *N*-arylimines to afford tetrahydroquinolines in addition to 4-aminobenzopyrans (Scheme 7).

Very recently, Melchiorre and co-workers successfully developed a vinylogous [4+2] Diels-Alder reaction with regard to the challenging tetrahydrocarbazoles, important structural units of natural products and pharmacologically active compounds [17].

**Scheme 8:**
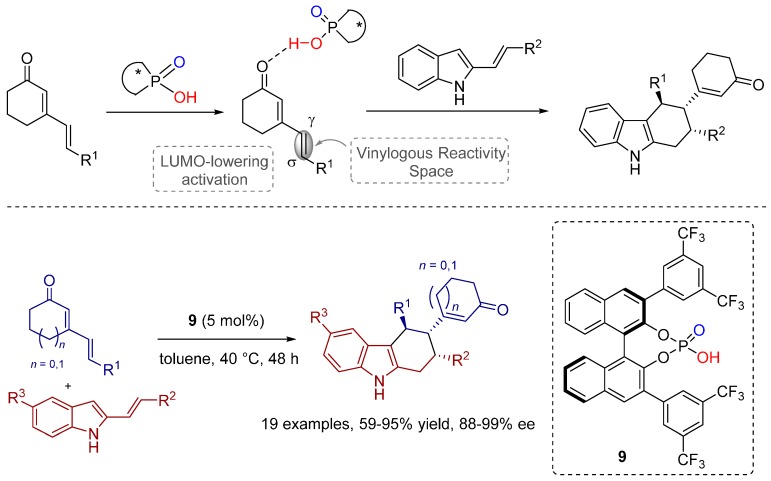
Vinylogous Diels-Alder reaction between cyclic 2,4-dienones and 2-vinylindoles.

In this context, cyclic 2,4-dienones have been proven to be suitable dienophiles for the synthesis, applying 2-vinylindoles as electron-rich dienes, and using chiral phosphoric acids as catalysts (Scheme 8). The variety of the substrate scope indicates the high potential and generality of the reaction.

Remarkably, the products were formed exclusively as single diastereomers with good to excellent enantioselectivities.

**Figure 7:**
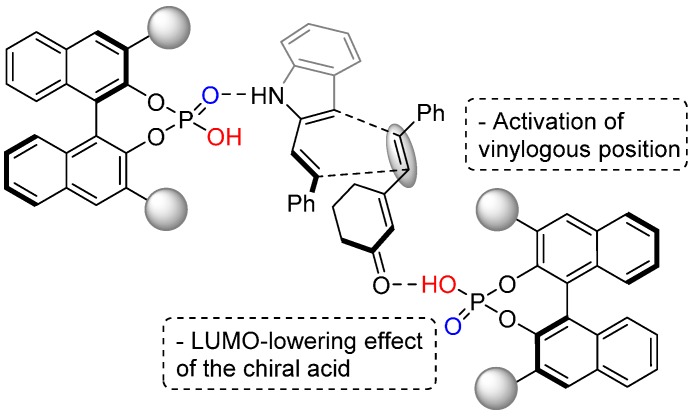
LUMO-lowering activation of the more distant double bond of α,β,γ,δ-unsaturated cyclic ketones.

Based on an observed positive nonlinear effect ((+)-NLE), a mechanistic model was proposed, accounting for the involvement of more than one molecule of the chiral phosphoric acid in the transition state structure (Figure 7).

Melchiorre was the first to use this specific activation principle where the vinylogous position of the dienophile is activated by the LUMO-lowering effect of the BINOL-phosphate, coordinating to the distant unsaturated cyclic ketones. To achieve high enantioselectivity, the requirement of an additional interaction between the Brønsted basic (P=O) moiety of the BINOL-phosphate catalyst and the secondary amine group of the diene has also been demonstrated experimentally.

### 2.4. 1,3-Dipolar Cycloadditions

**Scheme 9:**
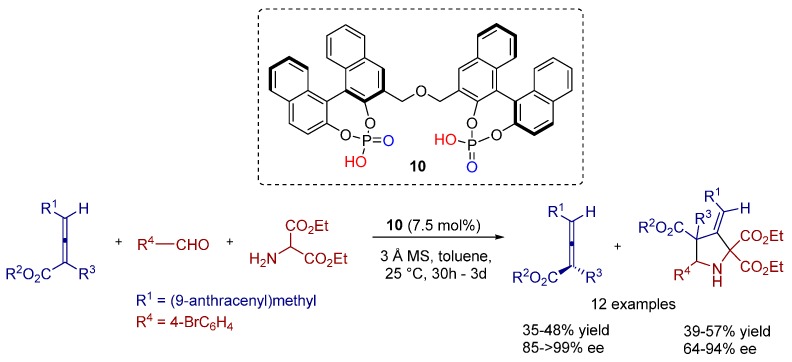
Kinetic resolution of racemic 2,3-allenoates by 1,3-dipolar cycloaddition catalyzed by chiral bisphosphoric acid.

In 2010, Gong and co-workers developed a kinetic resolution of racemic 2,3-allenoates via 1,3-dipolar cycloaddition by using bisphosphoric acid catalyst **10**. This method enables convenient access to both 2,3-allenoates and 3-methylenepyrrolidine derivatives, which are important precursors and/or subunits of biologically active compounds (Scheme 9) [18].

Thus, the key benefit of the developed method is the simultaneous generation of two versatile classes of compounds (3-methylenepyrrolidines and axially chiral 2,3-allenoates) in excellent enantiomeric excesses.

**Scheme 10:**
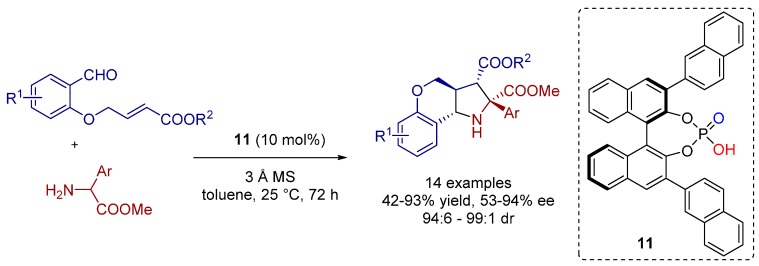
1,3-Dipolar cycloaddition between 4-(2-formylphenoxy)butenoates and α-aryl amino esters.

The same research group disclosed an elegant chiral Brønsted acid-catalyzed 1,3-dipolar cycloaddition of α-aryl amino esters with aldehydes bearing dipolarophile functionalities towards hexahydrochromeno[4,3-*b*]pyrrolidine derivatives with high levels of enantioselectivity (Scheme 10) [19]. The reaction outcome depends on the nature of the α-arylglycine methyl esters and the aldehydes, respectively. For instance, the use of α-arylglycine methyl esters with electron withdrawing aryl substituents is positively correlated to high levels of enantioselectivity.

**Scheme 11:**
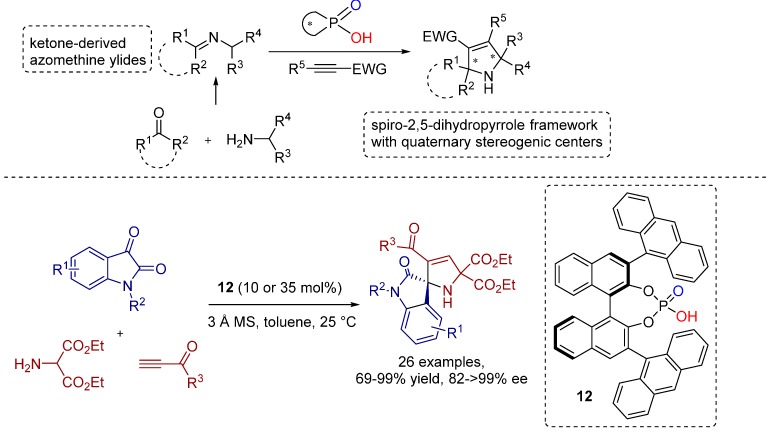
1,3-Dipolar cycloaddition of alkynes with isatin-derived azomethine ylides.

In 2013, Shi, Tu and co-workers reported the first catalytic asymmetric 1,3-dipolar cycloaddition of alkynes with isatin-derived azomethine ylides using chiral Brønsted acid catalyst **12** providing biologically important spiro-oxindole-based 2,5-dihydropyrrole frameworks (Scheme 11) [20].

**Figure 8:**
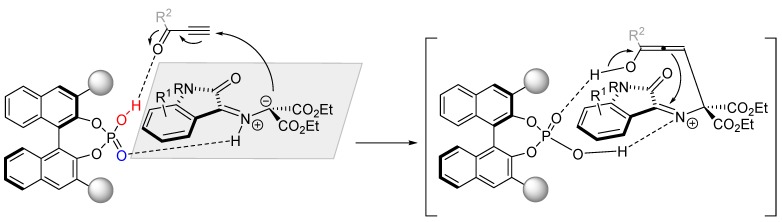
Proposed mechanism of 1,3-dipolar cycloaddition of alkynes with isatin-derived azomethine ylides.

Based on the obtained experimental results, the mechanism of BINOL-phosphate catalyzed 1,3-dipolar cycloaddition was proposed to proceed via sequential Michael addition and Mannich-type cyclization rather than via a concerted pathway (Figure 8).

The broad reaction scope clearly discloses the generality of this method. The catalyst performance does not depend on the substituents of the substrates and, hence, the pharmaceutically and synthetically important spiro-oxindoles were generated by this straightforward method in good to high yields and with excellent levels of enantioselectivity.

**Scheme 12:**
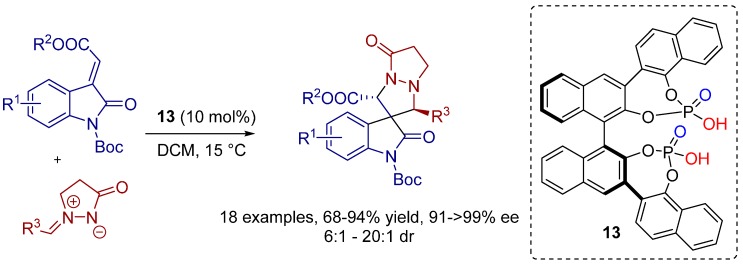
Enantioselective 1,3-dipolar cycloaddition between methyleneindolinones and *N*,*N*′-cyclic azomethine imines.

In 2013, Hong, Wang and co-workers developed a novel chiral bisphosphoric acid **13** bearing triple axial chirality as excellent catalyst for the generation of spiro[pyrazolidin-3,3′-oxindoles] via a 1,3-dipolar cycloaddition reaction of methyleneindolinones and *N*,*N*′-cyclic azomethine imine (Scheme 12) [22]. While some of the desired products were obtained with only moderate diastereoselectivity, the yields and enantioselectivities were excellent for a wide range of diverse substituted spiro-oxindoles.

**Figure 9:**
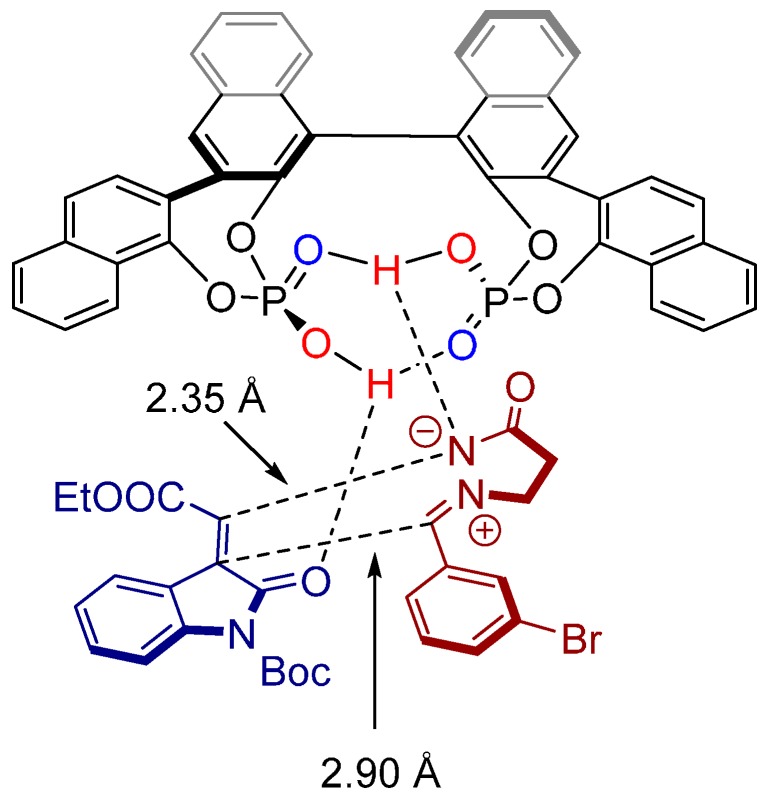
Transition state structure based on DFT calculations and MS experiments.

MS study and DFT calculations revealed a transition state structure wherein both the methyleneindolinones (dipolarophiles) and azomethine imines (1,3-dipoles) are activated simultaneously via hydrogen bonds through the OH groups of both chiral phosphoric acid moieties (Figure 9). This new activation mode may open unprecedented applications in other enantioselective organic transformations.

## 3. Cycloadditions Catalyzed by BINOL-(/SPINOL-)Derived *N*-Triflyl Phosphoramides

The relatively low acidity of BINOL-derived phosphoric acids limits the use of these organocatalysts to transformations requiring less acidic catalysts.

An approach to circumvent such limitation was earlier reported by the Koppel group via a modification of the acid moiety to increase its acidity. The effect of stepwise replacement of =O oxygen atoms by =NTf fragments in the sulfonyl group of toluene-*p*-sulfonamide and benzenesulfonamide on their acidity has been studied by Koppel and co-workers and it was demonstrated that the p*K_a_* of organic acids can be dramatically decreased and, thus, the activity of the corresponding catalyst can be raised [29,30].

**Figure 10:**
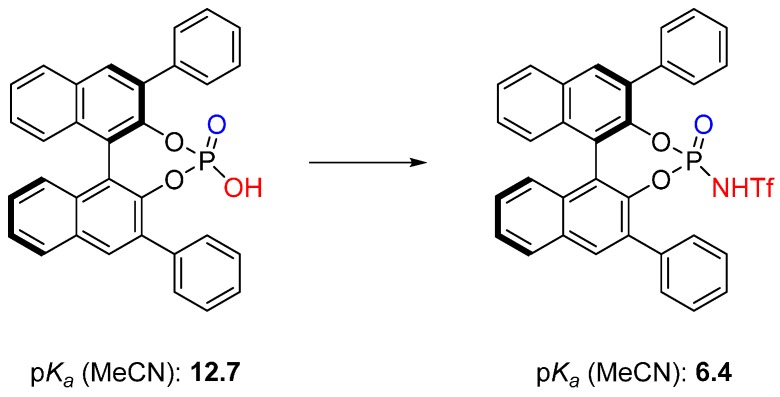
Acidity enhancement of BINOL-derived phosphoric acids via introduction of a NHTf-moiety [32].

This strategy was successfully employed for the first time in 2006 by Yamamoto and co-workers for the development of a highly enantioselective organocatalyst for the asymmetric Diels-Alder reaction [31]. Through the introduction of an NHTf-moiety into the BINOL-derived phosphoric acid the highly active and acidic chiral Brønsted acid catalysts were developed (Figure 10) [31].

Since then, many researchers have been encouraged to explore further asymmetric cycloaddition reactions catalyzed by highly acidic *N*-triflyl phosphoramide catalysts [32].

### 3.1. [3+2] Cycloadditions

**Scheme 13:**
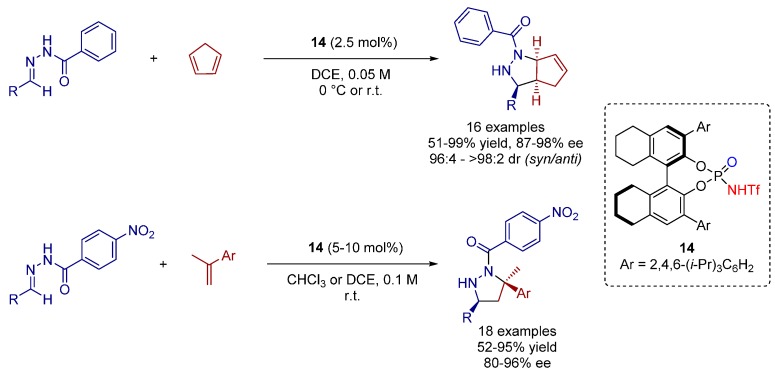
[3+2] Cycloaddition of *N*-acyl hydrazones to cyclic and terminal alkenes.

As a continuation of prior reported BINOL-phosphate catalyzed [3+2] cycloadditions towards pyrazolidines [14], Rueping and co-workers investigated the [3+2] cycloaddition of *N*-acyl hydrazones and different alkenes catalyzed by *N*-triflyl phosphoramides (Scheme 13) [33].

The more acidic, and, hence, active *N*-triflyl phosphoramides (p*K_a_* 6–7 in MeCN), compared to parent BINOL-derived phosphoric acids (p*K_a_* 12–14 in MeCN), promoted the cycloaddition reactions selectively towards the *syn* diastereomers as the major products providing fair to excellent yields, excellent diastereoselectivities throughout, and very good to excellent enantioselectivities.

When using unsubstituted *N*-acyl hydrazone, the catalyst loading can be reduced to 1 mol% with only slightly lower yield and enantioselectivity. The employment of further dienophiles was also investigated. A series of aromatic terminal alkenes was successfully converted to a variety of pyrazolidines bearing a quarternary and a tertiary stereocenter at the 3- and 5-positions with very good to excellent enantioselectivity.

**Scheme 14:**
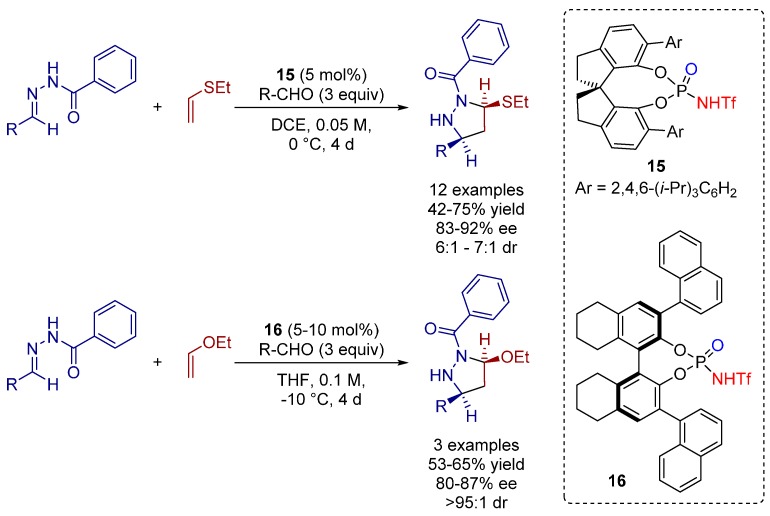
[3+2] Cycloaddition of hydrazones and alkenes catalyzed by *N*-triflyl phosphoramides.

The abovementioned approach towards enantiomerically enriched pyrazolidines was further pursued by the groups of Rueping and Houk in 2014. SPINOL-derived *N*-triflyl phosphoramide **15** was applied as a suitable catalyst for the [3^+^+2] cycloaddition between hydrazones and ethyl vinyl thioether towards a series of polysubstituted pyrazolidines with excellent enantioselectivity throughout (Scheme 14) [34].

Alternative ethyl vinyl ether was also well tolerated in the presence of *N*-triflyl phosphoramide catalyst **16** giving the corresponding adducts with enantioselectivity of up to 87%.

All mechanistic aspects of this reaction were supported by detailed computational calculations. The authors reached important conclusions concerning the mechanism of the cycloaddition by DFT (density functional theory) calculations.

**Figure 11:**
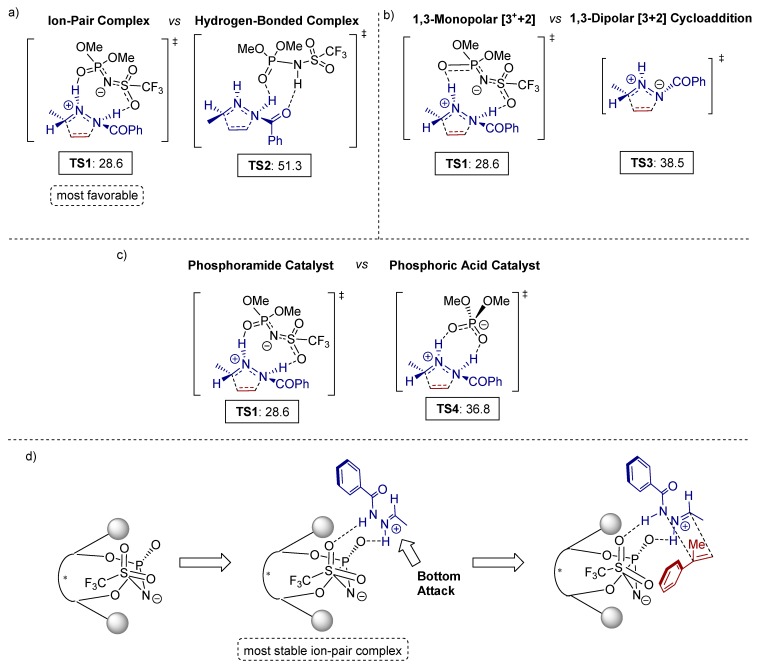
Mechanistic studies at DFT Level M06-2X/6-311+G(d,p) (energies in frames in Δ*G* = kcal/mol) using the conductor-like polarizable continuum model (CPCM) solvent model: A comparison of (**a**) two possible activation modes; (**b**) two possible reaction pathways; (**c**) phosphoramide and phosphoric acid catalyzed [3+2] cycloaddition; (**d**) activation and transition states for the enantioselective *N*-triflyl phosphoramide-catalyzed [3^+^+2] cycloaddition.

There are two possible pathways for the complexation of the hydrazone and the phosphoramide: either through proton transfer (ion-pair complex formation) or through hydrogen-bonding. Based on calculated Gibbs free energies, transition state **TS1** (Figure 11a), resulting from a proton transfer of the Brønsted acid to the hydrazone, is energetically most favorable among all transition states with ion-pair complexes. Additionally, a comparative energy profile of the monopolar [3^+^+2] and the competitive dipolar [3+2] cycloadditon revealed a lower overall barrier (28.6 kcal/mol) for the monopolar cycloaddition (Figure 11b). Thus, the proton transfer from the Bronsted acid to the hydrazone is essential for the catalytic efficiency, being therefore the predominant pathway. Furthermore, the obtained data demonstrate that the phosphoric acid is not capable of protonating the hydrazone due to its lower acidity compared to the *N*-triflyl phosphoramide analogues (Figure 11c), generating a hydrogen bonded-complex instead. To achieve the “ion pair”-geometry, necessary for the [3+2] cycloaddition transition state, a large distortion of the hydrogen-bonded complex is required, and, hence, the reaction barrier for this catalytic pathway turns out to be significantly higher (36.8 kcal/mol).

The high level of enantioselectivity is owed to the bulky substituents of the *N*-triflyl phosphoramide since it differentiates the stabilities of the possible conformers of the hydrazone-catalyst complex (Figure 11d). 

### 3.2. [4+2], Ionic [4+2] and Hetero [4+2] Cycloadditions

**Scheme 15:**
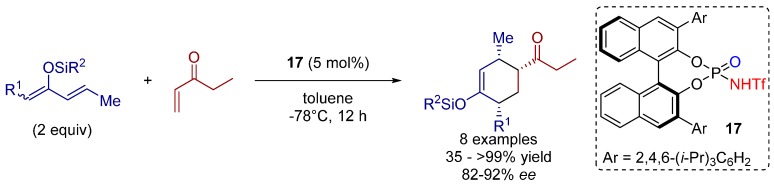
[4+2] Cycloaddition reaction of ethyl vinyl ketones and silyloxydienes.

In 2006, the first *N*-triflyl phosphoramide catalyst was developed by Yamamoto and co-workers for the asymmetric Diels-Alder reaction of ethyl vinyl ketone and different silyloxydienes. The reaction is highly *endo*-selective and the cyclohexenes were obtained in moderate to excellent yields and good to excellent enantioselectivities (Scheme 15) [31].

A low loading of the highly acidic and active catalyst **17** was sufficient for the generation of diversely substituted products. The bulkiness of the silyl moiety had no influence on the enantioselectivity, whereas the yields were quite sensitive to the stability of the silyloxydienes due to a possible deactivation of the catalyst by silylation.

**Scheme 16:**
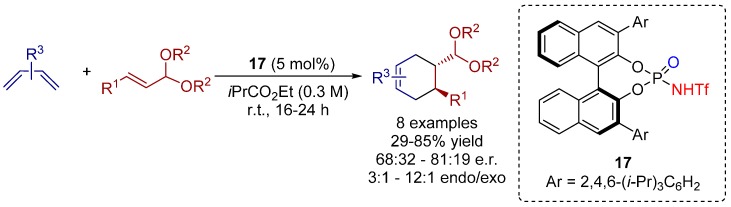
*N*-triflyl phosphoramide-catalyzed ionic Diels-Alder reaction between α*,*β-unsaturated acetals and different dienes.

In 2013, Nagorny and co-workers described the first example of an asymmetric chiral *N*-triflyl phorsphoramide-catalyzed ionic [4+2] cycloaddition reaction between α,β-unsaturated acetals and different dienes (Scheme 16) [35].

**Scheme 17:**
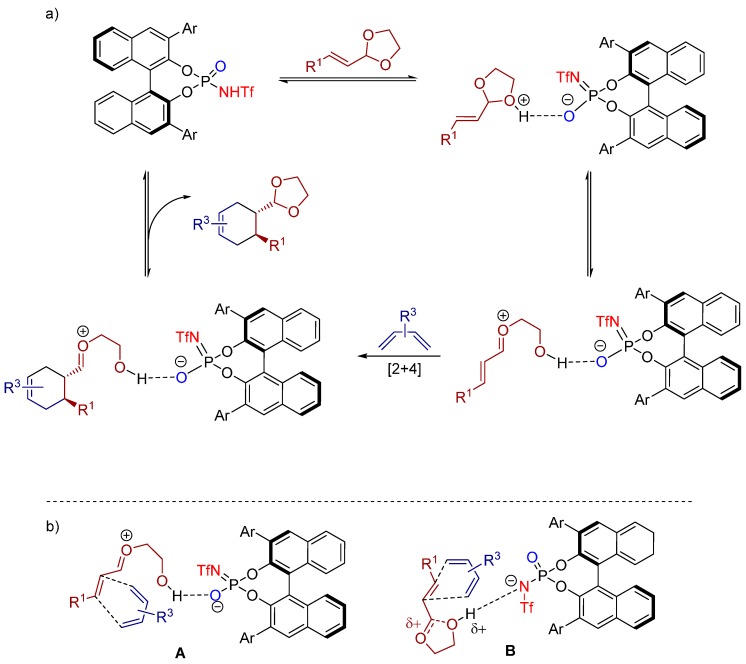
(**a**) Proposed reaction mechanism; (**b**) two possible transition states for the reaction.

It was shown, that the reaction rate and selectivity of the catalytic asymmetric ionic cycloaddition was strongly affected by the changes in the acetal portion of the dienophile. Moreover, the authors proposed a reaction mechanism for the ionic Diels-Alder reaction. The unsaturated acetals are, in an initial step, protonated by the Brønsted acid, forming a chiral ion pair in a reversible way, comprising the activated vinyl oxocarbenium cation which performs the [4+2] cycloaddition with the corresponding diene (Scheme 17a).

Furthermore, the authors showed two possible transition states (Scheme 17b). It was assumed that the reaction could proceed either through a defined oxocarbenium ion-like transition state **A** or through a transition state **B** wherein the cycloaddition step is coupled with the breakage of the C-O bond. A complete clarification of the reaction mechanism, however, can be achieved via computational studies.

**Scheme 18:**
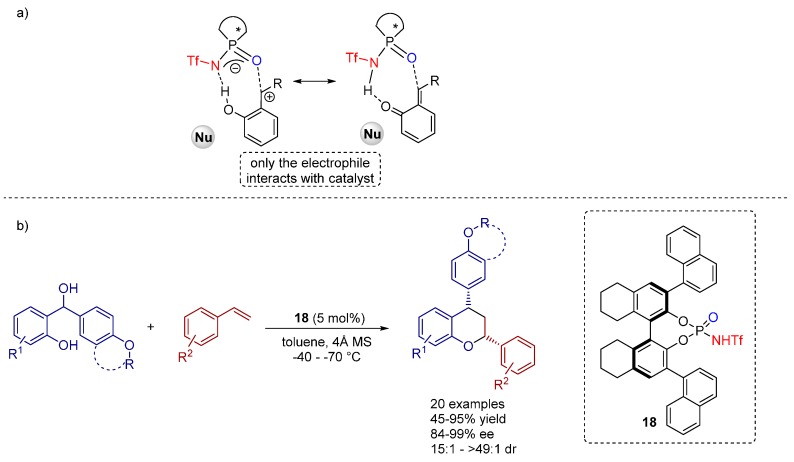
(**a**) The *ortho*-quinone methides as reactive intermediates; (**b**) [4+2] cycloaddition reaction towards chiral chromanes.

Recently, the Rueping group reported the Brønsted acid catalyzed [4+2] hetero-Diels-Alder reaction between unactivated alkenes and *in situ* generated *ortho*-quinone methides (from *ortho*-hydroxybenzyl alcohols) for the synthesis of chiral chromanes bearing multiple stereogenic centers with excellent diastereo- and enantioselectivities by the aid of a *N*-triflyl phosphoramide catalyst **18** (Scheme 18). Remarkably, the reaction proceeds via an open transition state with an exclusive activation of the electrophile, whereas the nucleophile does not interact with the catalyst [36].

A large substrate scope gives prove of the generality of the cycloaddition reaction, tolerating diversely substituted styrenes with various electronic and steric characteristics. Hence, all products, bearing either electron withdrawing or donating groups, could be furnished with excellent yields and high levels of enantioselectivity and diastereomeric ratio.

**Figure 12:**
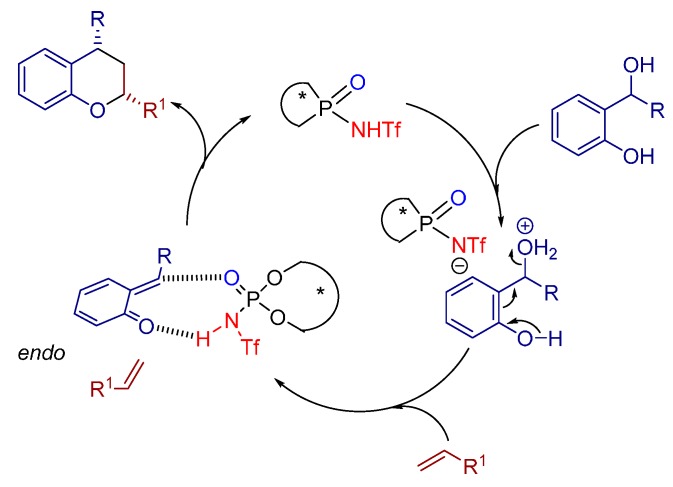
Proposed mechanism for the hetero Diels-Alder reaction, catalyzed by **18**.

The authors, moreover, proposed a reaction mechanism (Figure 12), further supported by NMR studies, which unveils the *in situ* generation of *ortho*-quinone methides (*o*-QM) via initial protonation of the corresponding *ortho*-hydroxybenzyl alcohol by the Brønsted acid. The catalyst then complexes the intermediary *o*-QM via hydrogen bonding and further stabilizes the methylene group of the *o*-QM with aid of the lone pair of the phosphoryl oxygen atom of the catalyst. In this transition state one face of the heterodiene is shielded by the catalyst allowing the alkene to attack selectively in an *endo* fashion.

### 3.3. 1,3-Dipolar Cycloadditions

**Scheme 19:**
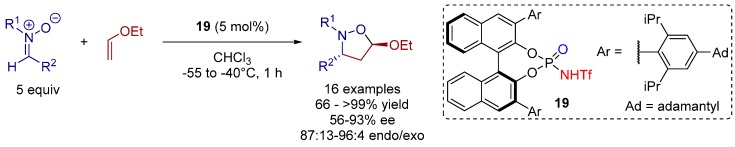
1,3-Dipolar cycloaddition of nitrones with ethyl vinyl ether.

In 2008, Yamamoto and co-workers carried out a 1,3-dipolar cycloaddition reaction catalyzed by a *N*-triflyl phosphoramide catalyst **19**. Within their studies they additionally emphasized the difference between Brønsted and Lewis acid catalysts (Scheme 19) [37].

Generally, the corresponding products were obtained in good to high optical purity and with good to excellent yields. The activity of the *N*-triflyl phosphoramide catalyst was demonstrated by rather short reaction times and the low catalyst loading needed for complete reaction.

**Figure 13:**
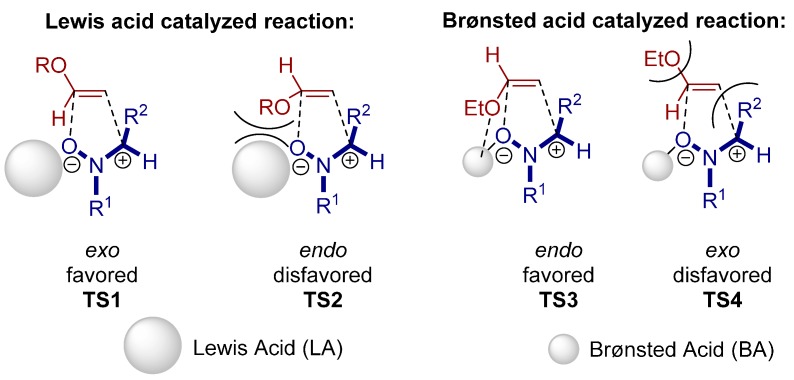
Transition-state structures showing the diastereoselectivity of the 1,3-dipolar cycloaddition of nitrones catalyzed by LA and BA.

The high *endo* selectivity of the reaction can be explained by the transition state (TS) structures. Due to steric repulsion between the ethoxy and R^2^ moieties in the Brønsted acid catalyzed *exo* TS4, the *endo* TS3 is energetically more favorable than TS4. In contrast, for a Lewis acid mediated reaction the *exo* approach (TS1) is preferred to the *endo* selective reaction (TS2) due to steric hindrance between the alkoxy group and the bulky Lewis acid in TS2 (Figure 13).

## 4. Conclusions

Over the past few years, a variety of organocatalyzed cycloaddition reactions have been developed and investigated in a number of laboratories, and significant progress has been made with chiral BINOL-derived organocatalysts. As seen in this review, chiral BINOL- and also SPINOL-derived *N*-triflyl phosphoramide and phosphoric acid catalyzed enantioselective cycloaddition reactions (e.g., [3+2]; formal [3+3]; [4+2]; vinylogous [4+2] and 1,3-dipolar cycloadditions) represent powerful transformations for the rapid and facile construction of bioactive heterocyclic compounds under mild conditions. Chiral Brønsted acids, known as privileged organocatalysts, and, in particular, recent novel chiral bisphosphoric acid catalysts are inspiring chemists to test further their full synthetic versatility.

The studies presented here add a new dimension to future investigations of applications of powerful chiral mono- and bisphosphoric acids, as well as *N*-triflyl phosphoramides in different pericyclic reactions and other new organic transformations and even in industrial-scale synthesis.
